# Genome-Wide Development of Polymorphic SNP Markers and Evaluation of Genetic Diversity of Litchi (*Litchi chinensis* Sonn.)

**DOI:** 10.3390/plants12233949

**Published:** 2023-11-23

**Authors:** Wei Liu, Zhidan Xiao, Nonghui Jiang, Chao Fan, Xu Xiang

**Affiliations:** Institute of Fruit Tree Research, Guangdong Academy of Agricultural Sciences, Key Laboratory of South Subtropical Fruit Biology and Genetic Resource Utilization, Ministry of Agriculture and Rural Affairs, Guangdong Provincial Key Laboratory of Tropical and Subtropical Fruit Tree Research, Guangzhou 510640, China; liuwei1@gdaas.cn (W.L.);

**Keywords:** litchi, germplasm resources, single-nucleotide polymorphism, genetic diversity, population structure

## Abstract

Litchi (*Litchi chinensis* Sonn.) is a highly valuable fruit crop that is widely grown in tropical and subtropical areas of the world. Studying its genetic diversity and population structure is critical for effective conservation and breeding programs. In this study, we developed 150 single-nucleotide polymorphism (SNP) markers that were evenly spaced across litchi genome and applied them to the evaluation of the genetic diversity of 84 litchi accessions, including old cultivars, modern cultivars, hybrids from known parents and wild accessions. Ninety-one SNP markers, showing high levels of polymorphism and high genotyping success rates, were used for further analysis. The newly developed SNP markers captured a relatively higher level of genetic diversity (He = 0.364) in litchi cultivars and could be successfully applied for the identification of synonymous cultivars and hybrids with close genetic backgrounds. Cluster analysis grouped all genotypes into three clusters that showed perfect association with their fruit maturation period, among which wild accessions clustered with their corresponding domesticated cultivars, and hybrids from different parent combinations showed different inheritance tendencies. Our study not only provided a set of efficient SNP markers for future genetic research, but also laid an important foundation for the conservation and genetic breeding of litchi.

## 1. Introduction

Litchi (*Litchi chinensis* Sonn.), a member of the family Sapindaceae, is a highly valuable fruit crop that is widely grown in tropical and subtropical areas of the world [1]. Litchi originated from China, where its domestication dates back ~2300 years [2], and has been introduced to other countries including India, Vietnam, Thailand, Madagascar, Australia etc., over the past 400 years [3]. Among these countries, China has the largest litchi industry in term of both cultivation area (490,933 ha) and total production (2,742,100 tons), accounting for 61.5% and 61.4% of the global cultivation area and production, respectively [4]. This fruit crop contributes significantly to the livelihood and economy of local people.

Due to the long history of litchi cultivation, abundant germplasm resources have been established in China, which contains the largest litchi germplasm gene bank in the world [5]. However, most cultivars ripen between mid-June and mid-July, leading to a centralized fruit maturation period [6,7]. Furthermore, pericarp browning and aril decay develop rapidly once the litchi fruit has been detached from the tree [8,9,10], resulting in an extremely short shelf life. Therefore, the breeding of novel litchi cultivars with more diverse fruit maturation periods (like extremely early or extremely late) and better storability after harvest, as well as improvement of other traits like fruit quality, disease resistance, adaptations to climate changes and etc., has become increasingly important for the sustainable development of the litchi industry in China [11].

However, the has been lots of confusion reported regarding litchi cultivar nomenclature among different litchi producing regions, which mainly results from misidentification, mislabeling, and the replacement of original cultivar names by local names [6]. Furthermore, detailed explorations of genetic relationships among litchi germplasm resources with enough material diversity and representativeness are currently scarce. These problems greatly hinder the process of genetic improvement of litchi in China.

The accurate identification of genotypes and comprehensive evaluation of genetic diversity and population structure of germplasm resources are crucial to breeding programs in terms of the proper selection of parent combinations and optimal exploitation of genetic resources [12,13,14,15]. Molecular genetic marker technology provides the most direct means of genetic relationship analysis [16]. These have been wildly applied to cultivar identification [17,18,19] and genetic diversity and population structure analysis [16,20,21,22,23]. Several types of molecular genetic makers have been used for genetic diversity analysis of litchi germplasm resources in China previously. For instance, Ding et al. used 37 random amplified polymorphic DNA (RAPD) markers to analyze the genetic diversity of 34 litchi cultivars collected in Fujian Province [24]. Chen et al. and Wang et al. used RAPD markers to investigate the genetic diversity of cultivars originating from Hainan province [25,26]. Liu and Mei used 30 RAPD makers to investigate the genetic relationships of 60 litchi cultivars in China, finding that litchi cultivars could be classified into three groups according to their fruit maturation periods [27]. Yi et al. and Peng et al. used amplified fragment length polymorphism (AFLP) markers to investigate genetic diversity of cultivars collected in Guangdong and Guangxi province, respectively [28,29]. Inter simple sequence repeats (ISSRs) and sequence-related amplified polymorphisms (SRAPs) were utilized to analyze genetic diversity of cultivars originating in Hainan and Guangdong Province, respectively [30,31]. Yao used 22 simple sequence repeat polymorphism (SSR) markers to analyze genetic diversity of 22 litchi accessions in Hainan Province [32]. Using 27 SSR markers, Fu found that 47 Chinese litchi accessions could be divided into three groups according to their fruit maturation periods [33]. In addition, based on 30 SSR markers developed from the expressed sequence tag, Xiang also found that 96 Chinese litchi accessions showed evidence of clustering according to fruit-maturation periods and geographic origin [34].

Single-nucleotide polymorphisms (SNPs) are more informative markers and have several advantages over other molecular markers, including being more frequent in genomes, mostly bi-allelic and highly reproducible among laboratories and detection techniques [35,36]. Additionally, they have been widely used for genetic diversity and population structure analysis in other fruit tree like citrus [23], grapevine [37,38], mango [22], pummelo [39] and pomegranate [40].

In our previous study, we investigated the genetic relationship of 96 litchi accessions in China using 90 SNP markers [7]. However, the studied accessions were mostly old litchi cultivars with an ancient history. There was a lack of modern cultivars newly bred in recent years, which have become the leading cross-parents in current litchi breeding programs in China. Wild accessions that are progenitors of cultivated litchi [1], serving as an important gene pool for the genetic improvement of litchi cultivars, were also not included. Furthermore, 90 SNP markers were selected based on the location of seventeen litchi linkage groups, which did not reflect the actual location at litchi chromosome. In 2022, chromosome-level genome assembly of litchi cultivar ‘Feizixiao’ was reported and a set of high-quality SNP loci was identified from resequencing data [1], providing rich genomic resources for SNP marker development. In this work, we aimed to (1) develop novel polymorphic SNP markers, evenly spaced across the litchi genome; (2) analyze the genetic diversity and population structure of litchi accessions including old cultivars, modern cultivars, hybrids from known parents, as well as wild accessions, which representing diverse genetic backgrounds. The findings of our study can eventually serve as an important basis for the genetic improvement and sustainable conservation of litchi germplasms resources in China.

## 2. Results

### 2.1. Development of Polymorphic SNP Markers for Litchi

**Figure 1:**
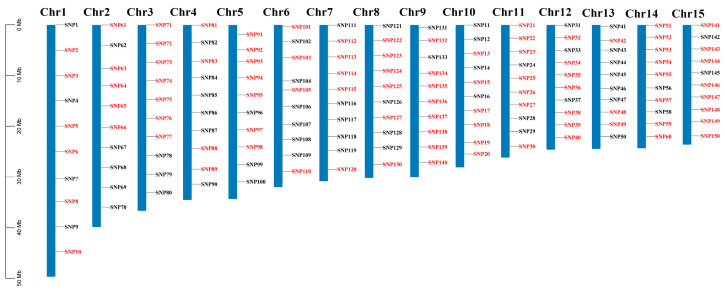
Schematic representations of the chromosomal location of 150 litchi SNP loci developed in this study. The 91 SNP loci retained for subsequent data analysis are denoted with red color, while the remaining SNPs are denoted with black color. The chromosome number is indicated on the top of each chromosome.

A set of 150 candidate SNP loci, evenly spaced across the 15 chromosomes of the litchi genome (Figure 1), were selected from SNP loci identified from litchi resequencing data [1]. The SNP name, chromosome location, and alternative alleles for the 150 SNP loci are listed in Table S1. These 150 SNP loci were then applied to genotype 84 litchi accessions. The accessions included 37 old cultivars, 18 modern cultivars, 12 hybrids from known parents and 17 wild accessions, representing a diverse genetic background. After filtering 1 locus unable to design genotyping primers, 28 loci that are monomorphic, 10 loci which have more than 10% missing data and 20 loci with a minor allele frequency (MAF) of less than 5%, 91 SNP loci were retained for the subsequent data analysis.

### 2.2. Genetic Diversity Analysis

**Figure 2:**
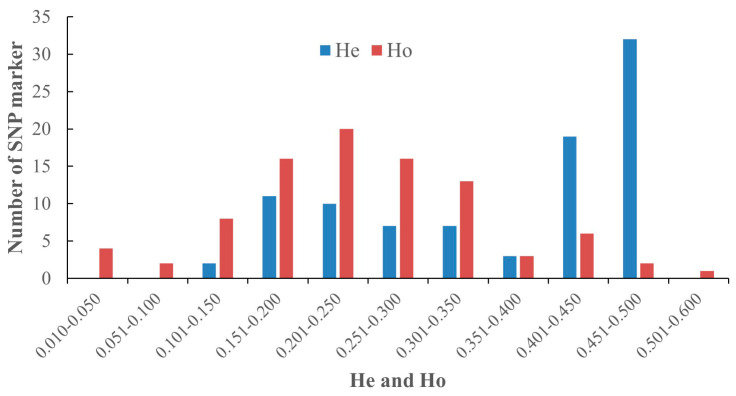
Distribution of expected heterozygosity (He) and observed heterozygosity (Ho) of 150 litchi SNP markers develop in this study.

Genetic diversity was investigated using the parameters of the expected heterozygosity (He) and observed heterozygosity (Ho). The He and Ho of the 91 SNP loci ranged from 0.107 to 0.500 and 0.000 to 0.690 (Figure 2). For the entire litchi collection, the average He and Ho values were 0.364 and 0.267, respectively (Figure 2). The same parameters were used to quantify the genetic diversity within different groups of old cultivars, modern cultivar, hybrids from known parents and wild accessions, which showed He of 0.371, 0.259, 0.348 and 0.365, respectively (Table 1). In general, broader genetic diversity was found within wild accessions and old cultivars.

### 2.3. Genetic Identification of the Litchi Collection

For genetic identification of the 84 litchi accessions, pairwise multilocus match analysis was applied among individual accessions using GenAlEx 6.5 [41]. The result identified five synonymous groups of litchi cultivars that showed identical genotypes based on the newly developed 91 SNP markers: ‘Sanyuehong’ from Guangdong Province and ‘Yuhebao’ from Guangxi Province; ‘Shuidong’ from Guangdong Province and ‘Nanxizaosheng’ from Taiwan Province; ‘Dazao’ from Guangdong Province, ‘Dahongpao’ from Sichuang Province and ‘Siyuehong’ from Guangxi Province; ‘Zengchengjinfeng’ from Guangdong Province and ‘Lanzhu’ from Fujian Province; and ‘Yuanhong’ and ‘Dachenzi’ from Fujian Province.

In addition to cultivar identification, the results also showed that ‘Guinuo 1’, ‘Guinuo 2’ and ‘Guinuo 3’, which are full siblings from same parents (‘Guiwei’ × ‘Nuomici’); ‘Zaogui’ and ‘Zaonuo’, which are half siblings from same maternal parent (‘Zaoli1hao’); as well as ‘Honggui’ and ‘Guihong’, which are hybrids derived from the reciprocal crossing of ‘Hongxiuqiu’ and ‘Guiwei’, could all be discriminated accurately based on genotypes on the newly developed 91 SNP loci, indicating a high discriminative power of our SNP marker set for hybrid identification of litchi.

### 2.4. Phylogenetic and Population Structure Analyses of the Litchi Collection

**Figure 3:**
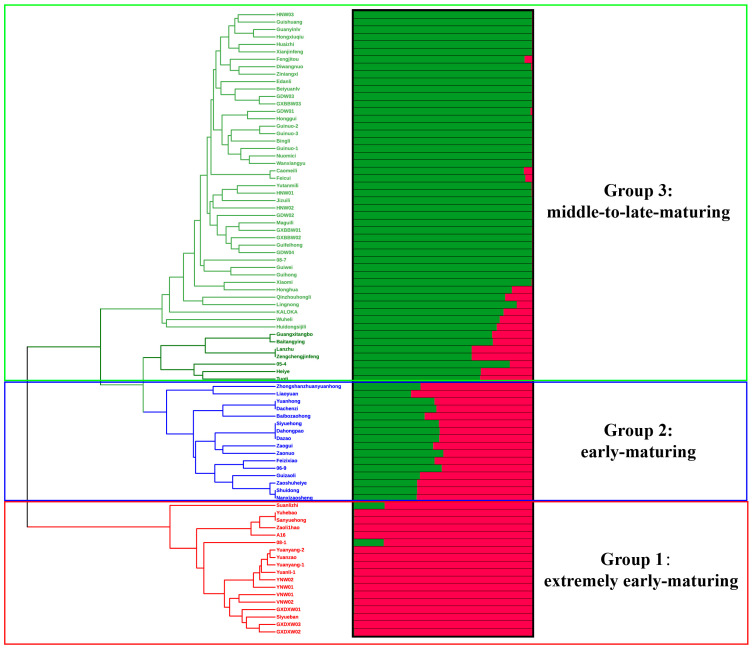
Phylogenetic tree and population structure of 84 litchi accessions based on 91 informative SNP markers. Phylogenetic tree was constructed with MEGA X using the unweighted pair-group method in combination with arithmetic mean (UPGMA) method. In the phylogenetic tree, red, blue and green color represent litchi accessions with extremely early-maturing period, early-maturing period and middle-to-late-maturing period, respectively. Population structure was analyzed using STRUCTURE 2.3.4. Structure bar plots of average proportions of membership for *K* = 2 clusters (red and green) are given for each of the 84 litchi accessions studied.

According to the phylogenetic tree constructed via the UPGMA method (Figure 3), the 84 litchi accessions were divided into three groups, which showed perfect association with their fruit maturation period: extremely early-maturing (EEM) group (Group 1), early-maturing (EM) group (Group 2) and middle-to-late-maturing (MLM) group (Group 3). Group 1 (n = 18) contained all EEM litchi cultivars like ‘Sanyuehong’, ‘Zaoli1hao’, ‘Yuanzao’ and ‘Yuanyang-1’, as well as wild Yunnan accessions (YNW), wild Guangxi accessions from Daxin County (GXDXW) and wild Vietnam accessions (VNM). In addition, ‘08-1’ which is hybrid progeny obtained from crossing YNW and old EM cultivar ‘Feizixiao’ and ‘A16’, which is hybrid progeny derived from crossing EEM cultivar ‘991’ and ‘Sanyuehong’, also clustered within Group 1. Group 2 (n = 16) included all EM cultivars like ‘Feizixiao’, ‘Shuidong’, ‘Dazao’ and ‘Guizaoli’. Progeny ‘06-9’, which is a hybrid derived from crossing old MLM cultivar ‘Heiye’ and old EM cultivar ‘Feizixiao’, clustered with its paternal parent ‘Feizixiao’ in Group 2. Furthermore, ‘Zaonuo’ and ‘Zaogui’, which are hybrids obtained using modern EEM cultivar ‘Zaoli1hao’ as the maternal parent and old MLM cultivar ‘Nuomici’ and ‘Guiwei’ as the paternal parent, respectively, were also clustered together in Group 2. Group 3 (n = 50) consisted of all MLM cultivars like ‘Guiwei’, ‘Nuomici’, ‘Xianjingfeng’ and ‘Huaizhi’, together with wild Guangxi accessions from Bobai County (GXBBW), wild Guangdong accessions (GDW) and wild Hainan accessions (HNW). Seven hybrid progenies (‘Guinuo 1’, ‘Guinuo 2’, ‘Guinuo 3’, ‘Honggui’, ‘Guihong’, ‘05-4’ and ‘08-7’), derived from combinations by using both MLM cultivars as maternal and paternal parents, were all clustered in Group 3. Interestingly, ‘Guinuo 1’, ‘Guinuo 2’ and ‘Guinuo 3’ which are full siblings from same parents (‘Guiwei’ × ‘Nuomici’), all clustered closely with their paternal parent ‘Nuomici’ rather than maternal parent ‘Guiwei’.

The population genetic structure was further analyzed using STRUCTURE 2.3.1 software [32]. The optimum number of clusters was recorded at *K* = 2 (Figure S1), indicating that there were two major clusters (Cluster I and II). The membership coefficient (q) is presented in Figure 3 in comparison to the UPGMA dendrogram. For the 18 EEM accessions in Group 1, 16 accessions showed a q value higher than 0.99 for Cluster I, while the old EEM cultivar ‘Suanlizhi’ from Sichuang Province and ‘08-1’ (hybrid progeny derived from crossing YNW and old EM cultivar ‘Feizixiao’) only showed q values of 0.827 and 0.832 for Cluster I, respectively. For 16 EM accessions in Group 2, all accessions showed a mixed origin with q values lower than 0.7 in relation to any cluster, indicating a considerable level of heterogeneity within these accessions. Overall, 48 out of 50 MLM accessions in Group 3, except for ‘Lanzhu’ (0.661) and ‘Zengchengjinfeng’ (0.661), were assigned to Cluster II with q > 0.7, among which 37 accessions had q > 0.99 for Cluster II. 

**Figure 4:**
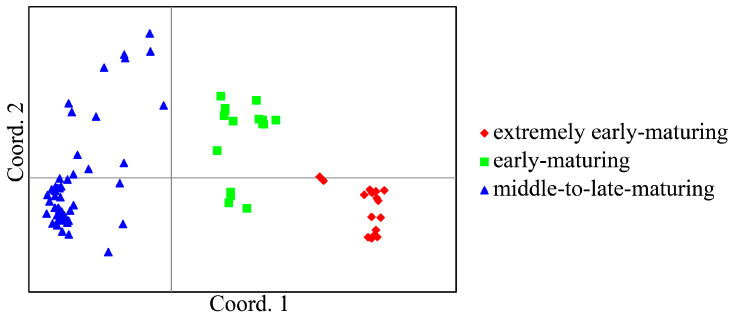
Principal coordinate analysis (PCoA) of 84 litchi accessions based on SNP markers. PCoA were performed based on standardized covariance of genetic distances calculated using GenAlEx 6.503. Red diamond, green square and blue triangle represent accessions with extremely early-maturing, early-maturing (EM) and middle-to-late-maturing fruit maturation period, respectively.

In addition, principal coordinate analysis (PCoA) was also performed, and we separated 84 litchi accessions into three groups in accordance with their fruit maturation period. This was consistent with the result of UPGMA clustering analysis (Figure 4). PC 1 accounted for 57.64% of the genetic variance and separated the EEM and EM group from the MLM group. PC 2 accounted for 3.91% of the genetic variation, which further separated the EEM group from the EM group, as well as middle-maturing cultivars from late-maturing cultivars of the MLM group.

We also performed analysis of molecular variance (AMOVA) to explore genetic variation among the 84 litchi accessions. The accessions were divided into four groups based on origin (old cultivars, modern cultivar, hybrids from known parents and wild accessions) and three groups based on fruit maturation period (extremely early-maturing, early-maturing and middle-to-late-maturing group), respectively. The AMOVA results showed that the variation among origin only explained 5% (*p* < 0.001) of the total variation, while the variation among fruit maturation group explained 51% of the total variation (*p* < 0.001) (Table 2), confirming that the fruit maturation period is the major criterion when classifying litchi cultivars.

Genetic differentiation among groups of different origin (old cultivars, modern cultivar, hybrids from known parents and wild accessions) and fruit maturation period (extremely early-maturing, early-maturing and middle-to-late-maturing group) were also tested using F_ST_ statistics estimated from pairwise comparisons. As for four groups of different origin, the lowest genetic differentiation was found between modern cultivars and wild accessions (F_ST_ = 0.004), the highest genetic differentiation was found between the modern cultivars and old cultivars (F_ST_ = 0.118) (Table 3), and the average F_ST_ among four groups was 0.054. As for the three groups of different fruit maturation period, the lowest genetic differentiation was found between the EEM and EM group (F_ST_ = 0.283), the highest genetic differentiation was found between the EEM and MLM group (F_ST_ = 0.691) (Table 3), and the average F_ST_ among three groups was 0.441. Therefore, the F_ST_ analysis revealed an much higher level of genetic differentiation among groups of different fruit maturation period than those of different origin.

## 3. Discussion

### 3.1. Genetic Diversity in the Studied Litchi Collection

To promote litchi breeding, it was necessary to conduct a comprehensive survey of the genetic diversity of litchi germplasm resource at a genome-wide scale with molecular markers. However, previous studies generally used a limited number of molecular markers with unknown distributions at the genome [27,29]. In this study, we utilized the newly reported SNP loci from a litchi genome resequencing study [1] and developed 150 SNP markers, evenly spaced across the 15 chromosomes of litchi genome, for the first time. After genotyping 84 litchi accessions, including old cultivars, modern cultivars, hybrids from known parents and wild accessions, 91 SNP markers with high levels of polymorphism and high genotyping success rates were obtained. The genetic variation found among litchi cultivars (He = 0.358) was higher than that reported in our previous study (He = 0.305) using SNP markers developed based on the location of litchi linkage groups [33]. Higher diversity suggested that the newly developed SNP markers, distributed across all 15 chromosomes of the litchi genome, have advantages over previous markers in deciphering genetic diversity.

Genetic diversity was further compared among old cultivars, modern cultivars, hybrids of known parents and wild accessions, showed expected heterozygosity (He) values of 0.371, 0.259, 0.348 and 0.365, respectively. In general, cultivars may contain lower levels of genetic diversity than that of wild germplasm owing to domestication bottleneck and intensive artificial selection [42,43]. The relatively higher level of genetic diversity found in old cultivars than wild accessions in our study may partly attributed to the smaller sample size in wild accessions compared to old cultivars. In addition, the lowest level of genetic diversity was found in modern cultivars that have been newly bred in recent years. This is likely because of intensive artificial selection pressure in pursuit of larger fruit, higher yield and fruit quality. Therefore, the fully utilization of the rich genetic diversity of litchi germplasm resources in future breeding work is critical to avoiding the loss of genetic diversity and broadening the genetic background of modern cultivars.

### 3.2. Genetic Identification of the Studied Litchi Collection

Due to the rapid development of the litchi industry in China, the introduction of different litchi cultivars among different litchi-producing provinces is rather frequent [6]. Sometimes, the same litchi cultivar may have different names in different locations [6], a phenomenon called ‘synonyms’. This is also reported in many other crops like grapevine [34,35], pummelo [36], pomegranate [37], cassava [38], common bean [39] and ginkgo [40]. Synonyms lead to large problems regarding cultivar identification and confuse the proper selection of breeding parents. Therefore, accurate characterization of litchi cultivars using molecular markers is crucial for genetic improvement. In our study, five synonymous groups of litchi cultivars, which showed identical genotypes at the 91 SNP loci, were identified. Among these four synonymous groups, ‘Sanyuehong’ from Guangdong Province and ‘Yuhebao’ from Guangxi Province; ‘Shuidong’ from Guangdong Province and ‘Nanxizaosheng’ from Taiwan Province; ‘Dazao’ from Guangdong Province and ‘Dahongpao’ from Sichuang Province and ‘Siyuehong’ from Guangxi Province; as well as ‘Zengchengjinfeng’ from Guangdong Province and ‘Lanzhu’ from Fujian Province were in agreement with expectations. This was because the synonymous cultivars in other provinces like Guangxi, Sichuan, Fujian and Taiwan Province were all introduced from Guangdong Province [2], which has the most abundant germplasm resources in China [6]. These synonymous groups have been suspected to be the same cultivars for many years due to having the same morphological and biological characteristics. Our results demonstrated that they are indeed synonyms and we avoided further confusion regarding their proper utilization in the breeding program. The remaining group of ‘Yuanhong’ and ‘Dachenzi’ from Fujian Province was unexpected, as they are both old cultivars originating in Fujian Province and were supposed to be two different cultivars [2]. Therefore, we speculated misidentification or mislabeling might have occurred during the introduction of these two cultivars from Fujian Province to Guangdong Province. 

In addition to cultivar identification, molecular markers also play important roles in hybrids identification [42,44,45]. Our results showed that the newly developed 91 SNPs could accurately discriminate full siblings from same parents (‘Guinuo 1’, ‘Guinuo 2’ and ‘Guinuo 3’), half-siblings from same maternal parent (‘Zaogui’ and ‘Zaonuo’), as well as hybrids from reciprocal crossing (‘Honggui’ and ‘Guihong’), indicating their high discrimination power. Therefore, the newly developed SNP marker set provides efficient molecular markers for cultivar and hybrid identification in litchi breeding work. 

### 3.3. Genetic Relationships and Population Structure among Litchi Accessions

Assessing the genetic relatedness and population structure of germplasm resources is essential for allowing breeders to identify optimal parental combinations [18,46,47,48,49]. In the present study, UPGMA, structure and PCoA analysis were performed in combination to detect the genetic relatedness and population structure in litchi germplasm resources. The UPGMA results indicated that the 84 litchi accessions were divided into three groups, which showed perfect association with fruit maturation period: extremely early-maturing (EEM), early-maturing (EM) and middle-to-late (MLM) group. The STRUCTURE analysis revealed the presence of two clusters (Cluster I and II) among the studied litchi collection, with EEM and MLM accessions assigned to Cluster I and II with nearly total ancestry, while EM accessions showed mixed origins of both clusters. And the PCoA analysis results further supported the results of the UPGMA and STRUCTURE analyses. These results were inconsistent with those reported in our previous study [45] and studies of other researchers [26,33]. Therefore, the fruit maturation period could be used as the most reliable criterion for clarifying genetic relationships among litchi germplasm. AMOVA and genetic differentiation (F_ST_) analysis further confirmed that the majority of genetic variation was found among three groups with different fruit maturation periods. In our UPGMA dendrogram, the EEM group contained all EEM cultivars like ‘Sanyuehong’, as well as wild accessions of YNW, GXDXW and VNW. The MLM group consisted of all MLM cultivars like ‘Guiwei’ and wild accessions of GXBBW, GDW and HNW. These results were incongruent with the two independent domestication events for litchi cultivars with different fruit maturation period, which found that EEM cultivars was domesticated from YNW, while MLM cultivars was domesticated from HNW [1]. The independent domestication events from different groups of wild litchi accessions of EEM and MLM cultivars also explained their significant genetic differentiation well, being derived from different genetic backgrounds.

In addition to cultivars and wild accessions, some hybrids from known parents were also included in order to analyze their genetic relationships with their parents. Among these, ‘Guinuo 1’, ‘Guinuo 2’ and ‘Guinuo 3’, which are full siblings derived from crossing two old MLM cultivars ‘Guiwei’ and ‘Nuomici’, all clustered closely with their paternal parent ‘Nuomici’ rather than maternal parent ‘Guiwei’. However, ‘Zaonuo’ and ‘Zaogui’, which are hybrid progeny created using the same modern EEM cultivar ‘Zaoli1hao’ as the maternal parent and two different old MLM cultivars, ‘Nuomici’ and ‘Guiwei’, as paternal parents, respectively, both showed closer genetic relationships with their maternal parent ‘Zaoli1hao’. We speculated the different inheritance tendencies of hybrids from different combinations may owe to the genetic relationships of parents because ‘Guiwei’ and ‘Nuomici’ both belong to MLM group, while ‘Zaoli1hao’ belongs to the EEM group which showed relatively high genetic differentiation (F_ST_ = 0.691) with the MLM group. Similar results have also been reported in other genetic diversity analyses of F1 hybrid populations of litchi using EST-SSR markers, which found that the genetic distance between cross-parents may affect the inheritance tendency and hybrid progeny from more genetically distant parents. Additionally, litchi showed closer genetic relationships with maternal rather than paternal parent [50]. As the number of hybrids used in this study was rather limited, future studies using lager hybrid populations are needed to further explore the inheritance pattern of hybrids in litchi to provide further support for optimal parental selection for the genetic improvement of litchi. 

## 4. Materials and Methods

### 4.1. Plant Material and DNA Extraction

Eighty-four litchi accessions (Table 4), available at the National Lychee Germplasm Repository and experimental orchards at the Institute of Fruit Trees, Guangdong Academy of Agricultural Sciences, Guangzhou, China, were utilized in this study. This litchi collection consisted of 37 old cultivars, with 18 modern cultivars newly bred in the last 20 years, 12 hybrids from known parents, and 17 wild accessions. Fresh and healthy leaves were collected from each litchi accession, frozen in liquid nitrogen, and stored at −80 °C. Total genomic DNA was extracted from the frozen fresh leaves of each litchi accession using the modified CTAB method, as described in previous research [51]. 

### 4.2. SNP Selection and Genotyping

This study utilized the newly reported SNP loci from litchi resequencing research [1] as a basis for developing polymorphic SNP markers for litchi. Among the reported SNP loci, three criteria were used for SNP marker selection: (1) being evenly spaced across the 15 chromosomes of litchi genome; (2) having a lack of other SNP loci in their 150 bp upstream and downstream; and (3) possessing a minor allele frequency (MAF) > 5%. A total of 150 SNPs were chosen, with 10 SNPs per chromosome. The SNP name, chromosome location and alternative alleles for the 150 SNP loci are provided in Table S1. Genotyping primers for the chosen SNP loci were designed using MassARRAY platform (Agena Bioscience, San Diego, CA, USA) (https://www.agenabio.com/products/massarray-system/, accessed on 1 November 2023). One locus that could not design genotyping primers successfully was abandoned and the remaining 149 loci were genotyped using the MassARRAY platform (Agena Bioscience, San Diego, CA, USA) according to the manufacturer’s instructions (https://www.agenabio.com/wp-content/uploads/2015/05/Agena-Bioscience-MassARRAY-SystemBrochure-SYS000306.pdf, accessed on 1 November 2023) at BGI (Shenzhen, China). 

### 4.3. Data Analysis

GenAlEx 6.503 software [41] was used to assess genetic diversity, measured as the expected heterozygosity (He) and observed heterozygosity (Ho), and to detect synonymous groups using the pairwise multilocus match analysis. Phylogenetic analysis was conducted using MEGA X software [52], and evolutionary history was inferred based on the unweighted pair-group method with arithmetic mean (UPGMA) using the p-distance model. Population structure was examined using STRUCTURE 2.3.4 software [44]. The analysis was performed based on the admixture model and the correlated allele frequencies between populations, with values of K set from 1 to 10. Ten replicates run were performed for each K, with a burn-in length of 10,000 and a run length of 100,000 iterations. The approach suggested by Evanno et al. [53] was adopted to calculate the most likely value of K using ad hoc ΔK statistics. Each individual was assigned to a cluster according to their membership coefficient (q), and the graphical bar plot of membership coefficients was generated using DISTRUCT 1.1 software [54]. To further infer genetic structure, principal coordinate analysis (PCoA) was also performed based on standardized covariance of genetic distances, which was calculated using GenAlEx 6.503 software. The analysis of molecular variance (AMOVA) was performed to determine internal and between-group variation by decomposing the 84 litchi accessions into four different groups by origin (old cultivars, modern cultivars, hybrids from known parents and wild accessions) and three different groups by fruit maturation period (extremely early-maturing, early-maturing and middle-to-late maturing group) using GenAlEx 6.503. The variance components were tested statistically via nonparametric randomization tests using 9999 permutations. The genetic differentiation between four different groups by origin (old cultivars, modern cultivars, hybrids from known parents and wild accessions) and three different groups by fruit maturation period (extremely early-maturing, early-maturing and middle-to-late maturing group) was analyzed via F_ST_ statistics using Arlequin 3.5 software [55].

## 5. Conclusions

In conclusion, this is the first study to develop a novel set of SNP markers that were evenly spaced across the litchi genome. We successfully applied them to genetic diversity and population structure analysis of litchi accessions with diverse genetic backgrounds. Our results demonstrate that the newly developed SNP markers are able to capture a relatively higher portion of genetic diversity compared to SNP markers with unknown locations in the genome. The SNPs set could also be effectively used for both cultivar and hybrid identification. Clustering analysis based on the SNPs confirmed that litchi accessions could be divided into three main clusters showing perfect association with their fruit maturation period, among which wild accessions clustered with their corresponding domesticated cultivars, and hybrids from different parent combinations showed different inheritance tendencies. The SNP markers developed in this study provide a valuable tool for future population genetics studies and breeding programs.

## Figures and Tables

**Table 1:** Genetic diversity patterns of old cultivar, modern cultivars, hybrids and wild accessions of litchi.

	Number of Accessions	He	Ho
Overall	84	0.364	0.267
Cultivars	55	0.358	0.278
(1) Old cultivars	37	0.371	0.322
(2) Modern cultivars	18	0.259	0.187
Hybrids	12	0.348	0.275
Wild accessions	17	0.365	0.127

**Table 2:** Analysis of molecular variance (AMOVA) among origin and fruit maturation period.

Source of Variation	Origin			Fruit Maturation Period		
	df	SS	%	df	SS	%
Among Pops	3	169.666	5%	2	1118.304	51%
Among Indiv	80	1807.197	33%	81	858.559	0%
Within Indiv	84	930.500	63%	84	930.500	49%
Total	167	2907.363	100%	167	2907.363	100%

Note: df: degree of freedom; SS: sum of squares; %: percentage of variation.

**Table 3:** F_ST_ among groups of different origin and fruit maturation period.

Origin	Old Cultivars	Modern Cultivars	Hybrids	Wild Accessions
Old cultivars	-			
Modern cultivars	0.118	-		
Hybrids	0.035	0.031	-	
Wild accessions	0.106	0.004	0.027	-
Fruit maturation period	EEM group	EM group	MLM group	
EEM group	-			
EM group	0.283	-		
MLM group	0.691	0.348	-	

**Table 4:** List of 84 litchi accessions used in this study.

No.	Accession Name	Status	Geographic Origin	Maturation Period	No.	Accession Name	Status	Geographic Origin	Maturation Period
1	Sanyuehong	old cultivar	GD	EEM	43	Guanyinlv	modern cultivar	GD	MLM
2	Dazao	old cultivar	GD	EM	44	Beiyuanlv	modern cultivar	GD	MLM
3	Zhongshanzhuanyuanhong	old cultivar	GD	EM	45	Bingli	modern cultivar	GD	MLM
4	Shuidong	old cultivar	GD	EM	46	Lingnong	modern cultivar	GD	MLM
5	Feizixiao	old cultivar	GD	EM	47	Guishuang	modern cultivar	GD	MLM
6	Guiwei	old cultivar	GD	MLM	48	Maguili	modern cultivar	GD	MLM
7	Nuomici	old cultivar	GD	MLM	49	Hongxiuqiu	modern cultivar	GD	MLM
8	Huaizhi	old cultivar	GD	MLM	50	Fengjitou	modern cultivar	GD	MLM
9	Heiye	old cultivar	GD	MLM	51	Wanxiangyu	modern cultivar	GD	MLM
10	Zengchengjinfeng	old cultivar	GD	MLM	52	Diwangnuo	modern cultivar	GD	MLM
11	Huidongsijili	old cultivar	GD	MLM	53	Caomeili	modern cultivar	GX	MLM
12	Baitangying	old cultivar	GD	MLM	54	Guifeihong	modern cultivar	GX	MLM
13	Yuhebao	old cultivar	GX	EEM	55	Yutanmili	modern cultivar	HN	MLM
14	Siyueban	old cultivar	GX	EEM	56	A16	hybrid	-	EEM
15	Siyuehong	old cultivar	GX	EM	57	08-1	hybrid	-	EEM
16	Zaoshuheiye	old cultivar	GX	EM	58	06-9	hybrid	-	EM
17	Guangxitangbo	old cultivar	GX	MLM	59	Zaogui	hybrid	-	EM
18	Qinzhouhongli	old cultivar	GX	MLM	60	Zaonuo	hybrid	-	EM
19	Jizuili	old cultivar	GX	MLM	61	Guinuo-1	hybrid	-	MLM
20	Yuanhong	old cultivar	FJ	EM	62	Guinuo-2	hybrid	-	MLM
21	Baibozaohong	old cultivar	FJ	EM	63	Guinuo-3	hybrid	-	MLM
22	Dachenzi	old cultivar	FJ	EM	64	Honggui	hybrid	-	MLM
23	Lanzhu	old cultivar	FJ	MLM	65	Guihong	hybrid	-	MLM
24	Edanli	old cultivar	HN	MLM	66	05-4	hybrid	-	MLM
25	Wuheli	old cultivar	HN	MLM	67	08-7	hybrid	-	MLM
26	Ziniangxi	old cultivar	HN	MLM	68	YNW01	wild accession	YN	EEM
27	Suanlizhi	old cultivar	SC	EEM	69	YNW02	wild accession	YN	EEM
28	Dahongpao	old cultivar	SC	EM	70	GXDXW01	wild accession	DX-GX	EEM
29	Tuoti	old cultivar	SC	MLM	71	GXDXW02	wild accession	DX-GX	EEM
30	Yuanli-1	old cultivar	YN	EEM	72	GXDXW03	wild accession	DX-GX	EEM
31	Yuanzao	old cultivar	YN	EEM	73	GXBBW01	wild accession	BB-GX	MLM
32	Yuanyang-1	old cultivar	YN	EEM	74	GXBBW02	wild accession	BB-GX	MLM
33	Yuanyang-2	old cultivar	YN	EEM	75	GXBBW03	wild accession	BB-GX	MLM
34	Nanxizaosheng	old cultivar	TW	EM	76	GDW01	wild accession	GD	MLM
38	Xiaomi	old cultivar	VN	MLM	77	GDW02	wild accession	GD	MLM
39	Honghua	old cultivar	VN	MLM	78	GDW03	wild accession	GD	MLM
40	KALOKA	old cultivar	TL	MLM	79	GDW4	wild accession	GD	MLM
35	Zaoli1hao	modern cultivar	GD	EEM	80	HNW01	wild accession	HN	MLM
36	Guizaoli	modern cultivar	GX	EM	81	HNW02	wild accession	HN	MLM
37	Liaoyuan	modern cultivar	YN	EM	82	HNW03	wild accession	HN	MLM
41	Xinjinfeng	modern cultivar	GD	MLM	83	VNW01	wild accession	VN	EEM
42	Feicui	modern cultivar	GD	MLM	84	VNW02	wild accession	VN	EEM

Note: For geographic origin, GD: Guangdong Province; GX: Guangxi Province; FJ: Fujian Province; HN: Hainan Province; SC: Sichuang Province; YN: Yunan Province; DX-GX: Daxin County of Guangxi Province; BB-GX: Bobai County of Guangxi Province; TW: Taiwan Province; VN: Vietnam; TL: Thailand. For maturation period, EEM: extremely early-maturing; EM: early-maturing; MLM: middle-to-late-maturing.

## Data Availability

The data contained within the present article and in its Supplementary Materials.

## Supplementary Materials

The following supporting information can be downloaded at: https://www.mdpi.com/article/10.3390/plants12233949/s1, Figure S1: DeltaK graph with optimal K; Table S1: Detailed information of 150 SNP loci used in this study.
