# Supplementary figures and images for: Genome-Wide Development of Polymorphic SNP Markers and Evaluation of Genetic Diversity of Litchi (Litchi chinensis Sonn.)
Source: Plants (Basel). 2023 Nov 23;12(23):3949. doi: 10.3390/plants12233949 (PMC10708096; doi:10.3390/plants12233949)

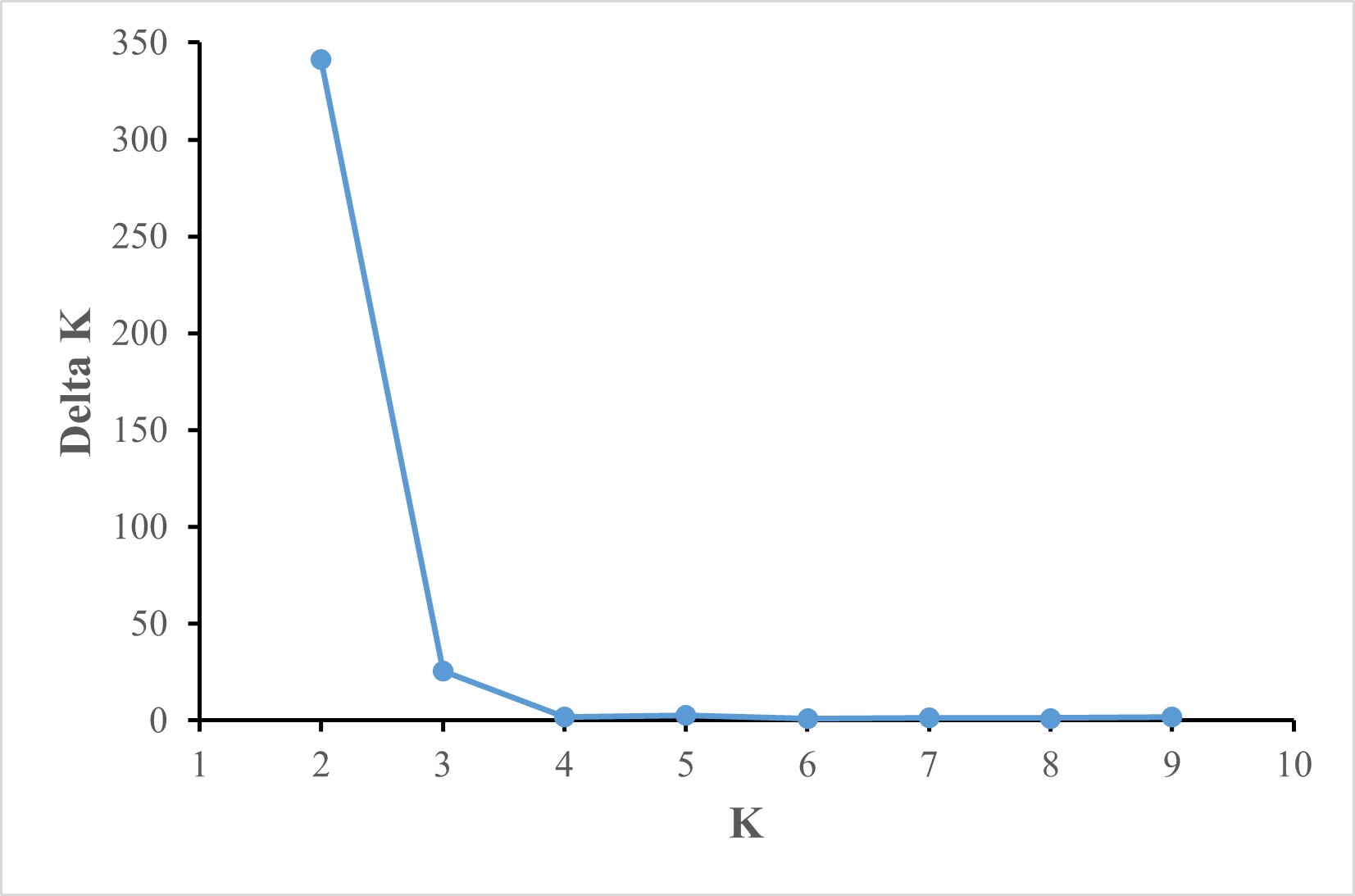

Supplement: Supplementary file 1 [file plants-12-03949-s001.zip › plants-2733630-supplementary/Figure S1 DeltaK graph with optimal K.jpg]
